# Recurrence-free survival and prognosis after adjuvant therapy with radioactive iodine-131 in patients with differentiated thyroid carcinoma

**DOI:** 10.1038/s41598-023-37899-z

**Published:** 2023-07-04

**Authors:** Yusuke Iizuka, Tomohiro Katagiri, Kengo Ogura, Minoru Inoue, Ryota Nakashima, Kiyonao Nakamura, Takashi Mizowaki

**Affiliations:** 1 Department of Radiation Oncology and Image-Applied Therapy, Kyoto University Graduate School of Medicine, 54, Shogoin Kawahara-cho, Sakyo-ku, Kyoto-shi, Kyoto 606-8507 Japan; 2 Department of Radiation Oncology, Shizuoka City Shizuoka Hospital, 10-93, Ote-machi, Aoi-ku, Shizuoka-shi, Shizuoka 420-8630 Japan

**Keywords:** Head and neck cancer, Radiotherapy

## Abstract

This study aimed to assess recurrence-free survival (RFS) rates and recurrence-related factors of patients who received adjuvant therapy (AT) with radioactive iodine (RAI) for differentiated thyroid cancer (DTC) following thyroidectomy. We evaluated 284 patients who underwent AT between January 2011 and July 2020 at our hospital. Recurrence was defined as visible recurrent lesions on image analysis or need for repeat surgery with pathologically confirmed recurrent lesions. RFS rate and prognostic factors were statistically evaluated. The median observation period was 30.2 months (range, 5.7–294 months). Overall, 192 patients were female and 92 were male, and the median age was 54 years (range, 9–85 years). Initial assessment revealed 39 recurrence cases. The 3-year RFS rate was 85.8% (95% confidence interval: 81.1–90.9%). Univariate analysis revealed that histology (except for papillary carcinoma), Tg level > 4 ng/dL before AT, and AT result significantly exacerbated the RFS rate. In multivariate analysis, histology and AT result were also important contributors to the worsening RFS rate. Results of AT can be determined relatively early and are important in predicting future recurrence in patients with DTC. Increasing the success rate of AT may lead to an improved prognosis.

## Introduction

Thyroid cancer is the most frequent cancer of the endocrine system, and its incidence is constantly rising worldwide. This is mainly because of advances in examination modalities that have made it possible to detect smaller cancers at an early stage. Owing to advancements in screening techniques and treatment methods, the number of patients who die from thyroid cancer is small compared to the incidence. Women are three times more likely to develop thyroid cancer than men^1^. The most common type of thyroid cancer is differentiated thyroid cancer (DTC), which includes papillary and follicular carcinomas. In Japan, the prevalence of DTC is reported to be more than 90%^2,3^.

The use of radioactive iodine (RAI) as part of radioisotope therapy for DTC following thyroidectomy, including “remnant ablation,” “adjuvant therapy (AT),” and “cancer treatment,” is well-accepted. AT is recommended, because of its therapeutic impact on micro-invasions or micro-metastases, to reduce the likelihood of recurrence in patients who do not have metastases but have a high risk of recurrence according to the Japanese guidelines and American Thyroid Association guidelines^4,5^. However, such situations cannot be easily identified. Ruel et al. reported that AT could prolong the survival of patients with intermediate-risk DTC^6^. Additionally, a meta-analysis has shown that AT is effective in reducing recurrence in some studies but not in others^7^, and the effect of AT on recurrence prevention, especially in high-risk DTC, remains unclear. One study has reported the death-suppressing effect of RAI administration in a high-risk patient group, but this report includes cases with metastasis and a high risk of recurrence, for which AT is not used^8^. In addition, it is not known whether the full benefits of AT can be obtained in individual patients or clinical settings.

Although there are reliable reports discussing the relationship between the success or failure of remnant ablation^9,10^, recurrence after AT is rarely reported and the number of cases is small^11,12^. In addition, no study has so far discussed the relationship between the success or failure of AT or remnant ablation and recurrence or death. No conclusions about the indicated doses for remnant ablation and AT have been drawn, with some studies showing no difference in success rates between high and low doses and others showing better results at higher doses^13–15^. The recommended dose for AT is stated in the guidelines but the statement is evaluated as “Weak recommendation, Low-quality evidence”^4,5^. Therefore, the aim of this study was to assess the recurrence-free survival (RFS) rate and recurrence-related factors, especially the relationship between RFS and AT dose, in patients who received AT with RAI after thyroidectomy.

## Results

The median observation period was 30.2 months (range, 5.7–294 months). The median age of the patients was 54 years (range, 9–85 years). The study included 192 female patients and 92 male patients. Doses of 1110, 1850, and 3700 MBq were administered to 111, 16, and 157 patients, respectively. Detailed characteristics are shown in Table 1.

**Table 1:** Patient characteristics.

Characteristics	N = 284
Sex	
Male	92 (32.4%)
Female	192 (67.6%)
Median age in years (range)	54 (9–85)
ECOG-PS	
0	167 (58.8%)
1	115 (40.5%)
≥ 2	2 (0.7%)
Histology	
Papillary carcinoma	269 (94.7%)
Others (follicular, poorly differentiated carcinoma, mixed)	15 (5.3%)
T stage	
T4	67 (23.6%)
T3	149 (52.5%)
T2	16 (5.6%)
T1	37 (13.0%)
T0	1 (0.4%)
Tx	14 (4.9%)
N stage	
N1b	176 (62.0%)
N1a	59 (20.8%)
N0	26 (9.2%)
Nx	23 (8.1%)
ATA risk^a^	
Low	0 (0%)
Intermediate	107 (62.3%)
High	177 (37.7%)
^131^I dose (MBq)	
1110	111 (39.1%)
2960	16 (5.6%)
3700	157 (55.3%)
Pre-Tg (mean, range) ng/mL	11.6 (0.80–488.5)

^a^ATA risk categories: low risk (pT1–pT2 and pN0), high risk (pT4, positive surgical margin, or extranodular invasion of any N stage), and intermediate risk (others). Pre-Tg indicates the level of serum thyroglobulin without TSH stimulation before the delivery of ^131^I.

*ECOG-PS* Eastern Cooperative Oncology Group performance status, *ATA* American Thyroid Association.

**Figure 1:**
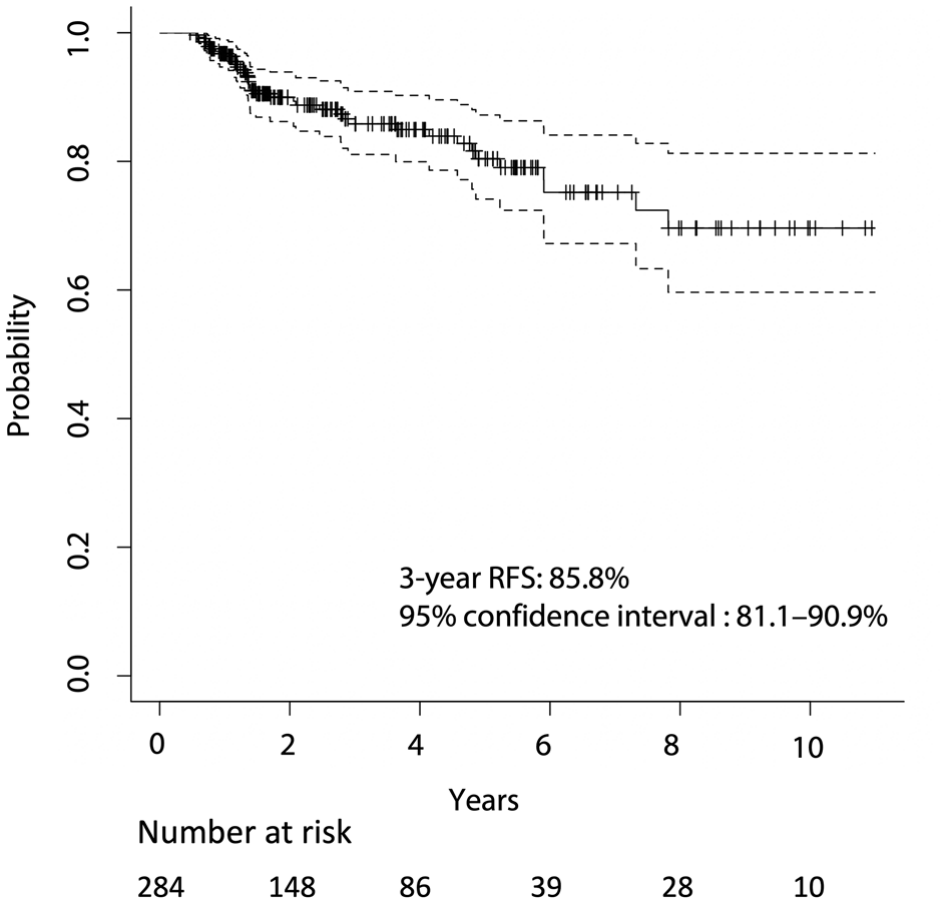
Kaplan–Meier curve of recurrence-free survival (RFS). Dotted lines indicate the 95% confidence interval.

Overall, 39 cases of recurrence were observed in the initial assessment. The following were the locations of the first recurrences: thyroid bed in two patients, cervical lymph nodes (LNs) in 16, mediastinal LNs in three, other LN sites in two, and lungs in 16 (including overlap). The 3-year RFS rate was 85.8% (95% confidence interval (CI): 81.1–90.9%; Fig. 1). Twenty-nine cases of recurrence were observed within 3 years.

**Figure 2:**
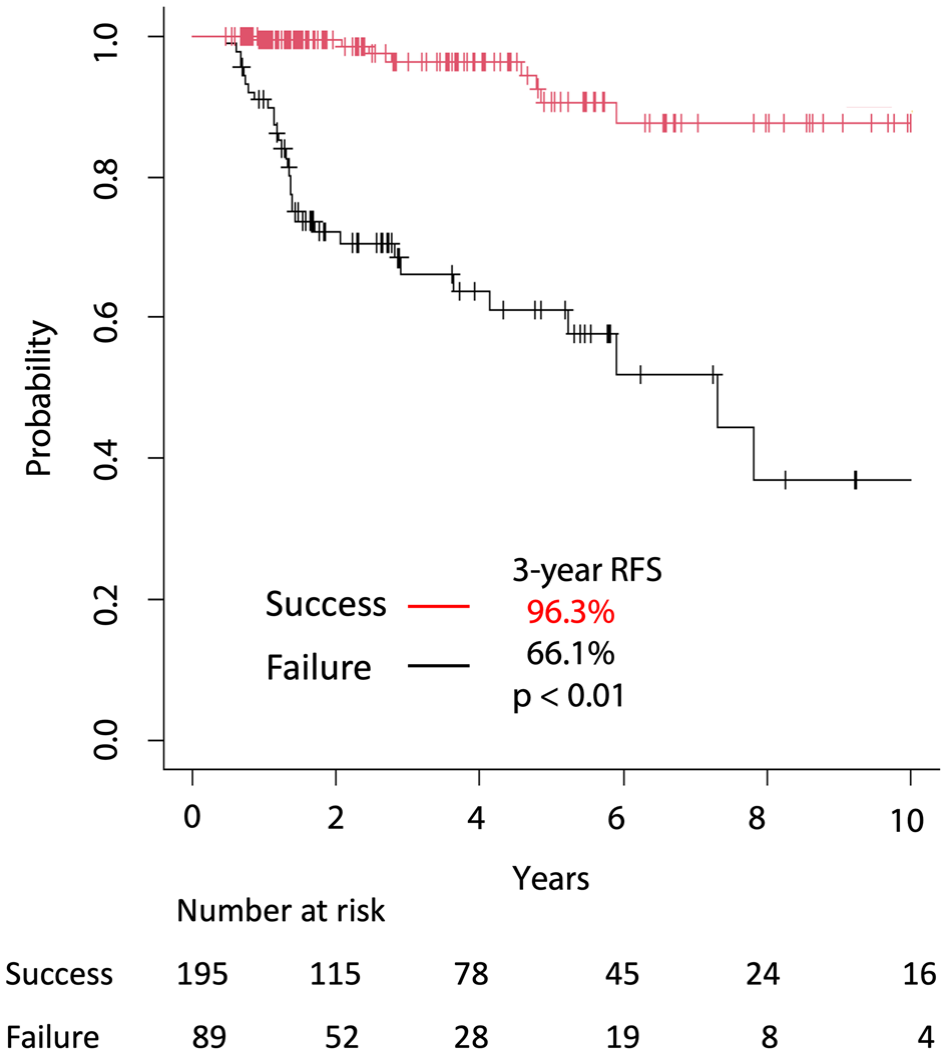
Kaplan–Meier curve of recurrence-free survival (RFS), stratified by success/failure of adjuvant therapy in all patients, with significantly more recurrence and death in patients who failed.

Univariate analysis revealed that histology (except for papillary carcinoma), Tg levels > 4 ng/dL before AT, and AT outcomes were significant factors that worsened the RFS rate. In Cox regression analysis, histology and AT findings were also important contributors to the worsening RFS rate (Table 2, Fig. 2). Two patients relapsed before the AT results were determined and were treated as failures for convenience in the aforementioned analysis. However, even when these two patients were excluded from the analysis, the AT result was a significant predictor of RFS in both univariate and multivariate analyses (p < 0.01). The median observation period of the AT success group was 28.6 months and that of the failure group was 33.2 months (p = 0.41). Although the low-dose group (1110 MBq) tended to have a better prognosis than the high-dose group (2960 and 3700 MBq), the difference was not significant (p = 0.07 in both univariate and multivariate analyses).

**Table 2:** Prognostic factors for recurrence or death. p-value was calculated with a log-rank test concerning the time period. *p-value calculated with logistic regression analysis.

N = 284	Recurrence or death			
Factors	+ (N = 40)	− (N = 244)	Univariate p-value	Multivariate p-value
Sex				
Male	18 (6.3%)	74 (26.1%)	0.07	
Female	22 (7.8%)	170 (59.9%)		
Age in years				
≥ 55	24 (8.5%)	114 (40.1%)	0.17	
< 55	16 (5.6%)	130 (45.8%)		
Histology				
Papillary carcinoma	35 (12.3%)	234 (82.4%)	< 0.01	0.02, *0.08
Others	5 (1.8%)	10 (3.5%)		
T stage				
T4	11 (3.9%)	56 (19.7%)	0.43	
T3	20 (7.0%)	129 (45.4%)		
T2	0 (0%)	16 (5.6%)		
T1	6 (2.1%)	31 (10.9%)		
T0	0 (0%)	1 (0.4%)		
Tx	3 (1.1%)	11 (3.9%)		
N stage				
N1b	28 (9.9%)	148 (52.1%)	0.39	
N1a	7 (2.5%)	52 (18.3%)		
N0	0 (0%)	26 (9.2%)		
Nx	5 (1.8%)	18 (6.3%)		
Pre-Tg^a^				
> 4 ng/mL	23 (8.1%)	51 (18.0%)	< 0.01	0.95, *0.46
≤ 4 ng/mL	17 (6.0%)	193 (68.0%)		
ATA risk				
Low	0 (0%)	0 (0%)	0.49	
Intermediate	22 (7.8%)	155 (54.6%)		
High	18 (6.3%)	89 (31.3%)		
Margin+ or ECI				
+	12 (4.2%)	52 (18.3%)	0.23	
−	28 (9.9%)	192 (67.6%)		
Dose (MBq)				
1110	11 (3.9%)	100 (35.2%)	0.07	0.07, *0.27
2750–3700	29 (10.2%)	144 (50.7%)		
AT result				
Success	8 (2.8%)	187 (65.8%)	< 0.01	< 0.01, *< 0.01
Failure	32 (11.3%)	57 (20.1%)		

^a^Pre-Tg indicates the level of serum thyroglobulin without TSH stimulation before the delivery of ^131^I. ATA risk categories: low risk (pT1–pT2 and pN0), high risk (pT4, positive surgical margin, or extranodular invasion of any N stage), and intermediate risk (others).

*ECOG-PS* Eastern Cooperative Oncology Group performance status, *ATA* American Thyroid Association, *ECI* extracapsular invasion of lymph nodes, *AT* adjuvant therapy.

**Figure 3:**
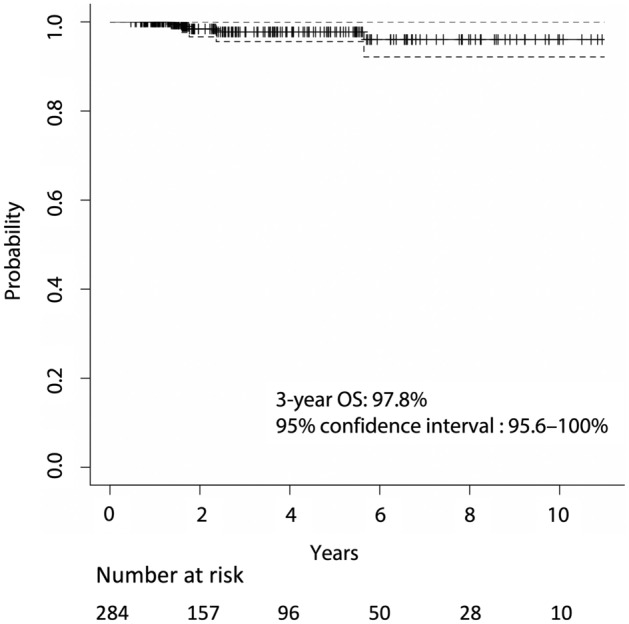
Kaplan–Meier curve of overall survival (OS). Dotted lines indicate the 95% confidence interval.

Five patients died during observation: four died from the primary disease and one died from other causes. The estimated OS was 97.8% (95% CI 95.6–100%) at 3 years (Fig. 3). The median observation period of the high-dose group was 31.0 months and that of the low-dose group was 27.6 months (p = 0.63). All the patients who died were from the high-dose group, and the estimated OS was 96.2% (95% CI 90.9–98.4%). In univariate analysis, histology (except for papillary carcinoma) (p < 0.01) and AT outcomes (p = 0.04) were significant factors that worsened the OS rate. In Cox regression analysis, histology (p < 0.01) and AT results (p = 0.05) were also statistically significant predictors of a worsening OS rate.

## Discussion

We retrospectively evaluated RFS rates and recurrence patterns after AT in patients with DTC. The 3-year RFS rate was 85.8% (95% CI 81.1–90.9%) and the most common recurrence site was cervical LNs.

Histology (except for papillary carcinoma, N = 15) was a significant poor prognostic factor for RFS. The breakdown of this group was as follows: follicular carcinoma, 7; poorly differentiated carcinoma, 7; and mixed papillary and follicular pattern, 1. A possible explanation for these observed results could be that poorly differentiated carcinoma has a poor prognosis^16,17^. AT success/failure is associated with Tg levels, and cases with a high pre-Tg level also have more recurrences. The pre-Tg level can imply the presence of residual lesions at the thyroid bed or distant metastases elsewhere after surgery, which may contribute to future recurrences. Patients whose metastases were identified by ^131^I scintigraphy during AT (excluded from this study) had higher Tg levels than the overall population (mean, 99.1; range, 0.25–429.6 ng/mL).

AT failure is significantly associated with recurrence. We analysed the prognostic factors for AT in a previous study and found that cases with Pre-Tg levels greater than 4 ng/mL had significantly more AT failures^18^. AT results can be evaluated within 1 year at most and may be useful as an early predictor of recurrence^9,10^. To date, there have been no reports of methods or favourable populations that increase the success rate of AT; therefore, careful follow-up is desirable for patients with failed AT. This is the first report to discuss the relationship between the success and failure status of AT and DTC recurrence. Criteria for successful AT have not been definitively established, and they tend to vary across different reports. For example, they are based on serum Tg concentration with thyroid-stimulating hormone (TSH) stimulation or non-stimulation, imaging studies (^131^I scintigraphy or ultrasonography), or a combination of these. Indeed, the definition of AT success in the present study is not always used, but we use the same criteria as in previous studies in order to be consistent in our discussion^18^: “no accumulation of ^131^I in the thyroid bed on scintigraphy and a serum Tg concentration < 2.0 ng/mL without TSH stimulation”. Radiation dose and positive surgical margins or extranodal invasion are sometimes said to be associated with recurrence^19,20^; however, in this study, there was no significant effect on RFS. Rather, RFS tended to be better in the low-dose group, which may have been influenced by the tendency to use high doses in high-risk cases. There are no studies on the appropriate doses in AT yet, but guidelines recommend doses up to 5550 MBq^4,5^. Reports of AT at even higher doses are awaited.

There are few reports on clinical outcomes after AT with RAI, particularly in patients at intermediate or high risk of recurrent DTC. Watanabe et al. reported the RFS rate after AT with microscopically positive tumour margins^11^. They evaluated 48 patients who underwent AT with low-dose (1110 MBq, N = 17) or high-dose (3700 MBq, N = 31) RAI after total thyroidectomy and reported nine cases (52.9%) of recurrence in the low-dose group and seven (22.5%) in the high-dose group. The 3-year RFS rates were approximately 85% in the low-dose group and 94% in the high-dose group. RFS in the low-dose group was significantly shorter than that in the high-dose group. Lymph node metastases were the most common type of recurrence. Jeong et al. reported the RFS rate in 253 patients with T4 or N1b DTC who underwent AT with different ^131^I doses (3700 MBq vs 5550 MBq). Of the 253 patients, 22 (8.7%) experienced recurrence after AT, and the high-dose and low-dose groups did not differ significantly from one another. The 3-year RFS rate was approximately 93%, with the most common type of recurrence being cervical LN metastasis. Our results were worse than those of the Korean study, and the cause of this is unclear. The authors stated that these patients were operated on by only two experienced surgeons at a high-volume tertiary care centre. This finding suggests that skilfully done surgery reduces the risk of recurrence. In addition, recurrence was histologically or cytologically proven, and it is possible that lung metastasis, which can be diagnosed only by imaging, had been overlooked. The primary endpoint of the study by Jeong et al. was the success or failure of AT, and the relationship between this and RFS was not described^12^.

The definition of recurrence is controversial, and the clinical diagnosis of recurrence is difficult. Lesions that take up iodine can be diagnosed with high sensitivity by ^131^I scintigraphy but those that do not take up iodine change a little over time and are often difficult to diagnose with imaging. ^18^F-Fluorodeoxyglucose positron emission tomography may be used in patients with negative ^131^I scintigraphy or anti-Tg antibodies^21,22^. Lymph node metastases may be pathologically diagnosed after neck dissection in patients with a high Tg level, but small pulmonary nodules may be indistinguishable from inflammatory changes. In this study, lung lesions were diagnosed using imaging, which was less accurate. An accurate diagnosis of non-iodine-uptake lesions is also needed.

This study had a few limitations. Firstly, it was an observational retrospective study conducted at a single institution. However, the treatment policy and dose were consistent, and the selection bias was small. Secondly, the observation period was short, and the sample size was small. Lastly, we observed only five cases of death, and we could not evaluate whether AT prolonged the OS. To assess the relationship between AT and OS rates, a long-term comparison study with a larger sample size is required.

In patients who received AT with RAI, the estimated 3-year RFS rate was 85.8%. Histological and AT results significantly influenced the RFS rate. The AT result can be determined relatively early and is an important index for predicting future recurrence. Thus, increasing the success rate of AT may lead to an improved prognosis.

## Materials and methods

### Study design

**Figure 4:**
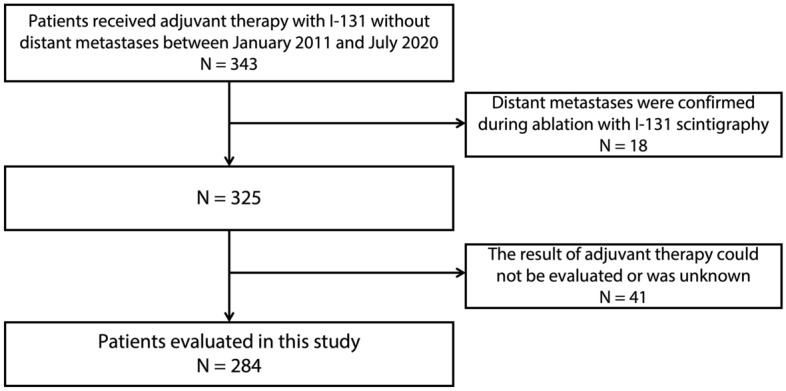
Patient selection for this study.

This retrospective evaluation was performed on patients who received AT in our hospital for DTC after surgical resection without macroscopic residual or metastatic lesions (patients with gross residual lesions at the tumour bed or at the site of lymph node resection who were considered to be receiving “cancer treatment” were not included in this study). A total of 343 patients underwent AT between January 2011 and July 2020. Of these, 284 patients were evaluated after excluding those whose metastases were confirmed during AT and those whose AT results were unknown (Fig. 4).

### Procedure for AT

Preparations for AT were as follows: iodine intake restriction for 2–3 weeks strictly and thyroid hormone restriction or use of recombinant human thyroid-stimulating hormone (rhTSH) to elevate the serum TSH level prior to ^131^I administration.

^131^I was administered in doses of 1110 MBq (low-dose group) or 2960–3700 MBq (high-dose group). Radiation oncologists recommend these doses based on pathological findings, ATA risk stratification, patient preference, and the environment of the patient’s residence. None of the patients were included in the ATA low-risk group, and all patients were treated with ^131^I as AT. In Japan, as only a dose below 1110 MBq of ^131^I is permitted for use in AT when administered in outpatient settings, outpatients who underwent AT were included in the low-dose group and inpatients were classified into the high-dose group. Three days after ^131^I administration, whole-body ^131^I scintigraphy was performed using a gamma camera with high-energy collimators (Infinia Hawkeye4, GE Healthcare, Milwaukee, WI, USA). Serum thyroglobulin (Tg) concentration was also determined. Three to 12 months after AT, a diagnostic ^131^I scintigraphy (370 MBq) was performed to determine whether AT was successful. According to our study and earlier investigations, initial AT success was defined as the absence of ^131^I accumulation in the thyroid bed on ^131^I scintigraphy and a serum Tg concentration of < 2.0 ng/mL without TSH stimulation. We used the same criteria as in our previous study^18^, determined with reference to previous reports^9,10^. Patients who tested positive for anti-Tg antibodies were determined based on their ^131^I scintigraphy results, whereas those who did not undergo ^131^I scintigraphy were evaluated based on serum Tg concentration. Eastern Cooperative Oncology Group performance status (ECOG-PS) was determined through a medical interview and physical examination at the first visit by the doctor in charge.

### Follow-up and outcome measures

Patients were examined every 2–6 months after AT, serum Tg concentration was measured every 3–6 months, and neck-to-chest computed tomography (CT) or neck ultrasonography was performed if the physician thought the examination was needed, until the end of follow-up. The last day of follow-up was the date of death or when survival was confirmed (mainly the date of the outpatient visit). Patients with visible recurrent lesions on image analysis or those who underwent repeat surgery with pathologically confirmed recurrent lesions were defined as having recurrent lesions. RFS was defined as the absence of recurrence or mortality from any cause, whereas OS was defined as mortality from any cause.

### Statistical analysis

R Software version 4.0.2 (R Foundation for Statistical Computing, Vienna, Austria) was used to perform all statistical analyses^23^. The Kaplan–Meier method was used to estimate RFS and OS rates, and the log-rank test was used to determine the contribution of each factor to RFS. Age, sex, histology, performance status, ATA risk classification, and thyroglobulin concentration all affected the therapy dose; hence, Cox regression analysis was performed to adjust the effect of these confounders. Because it is presumed that some cases may involve latent lesions that do not show the effect of AT at the time of AT and only become evident later, an additional multivariate analysis was performed that did not take the time factor into account. Statistical significance was set at p < 0.05.

### Ethics approval

The 1964 Declaration of Helsinki and all amendments thereto, as well as the recommendations of the Ethical Guidelines for Medical and Health Research Involving Human Subjects, served as the foundation for this retrospective observational study. The study was approved, and due to its retrospective nature, the need for informed consent was waived by Kyoto University Graduate School and Faculty of Medicine and Kyoto University Hospital Ethics Committee (R1087; April 19, 2017). The study was carried out utilising the opt-out procedure described on our hospital website.

### Consent to participate

Due to the retrospective nature of the study, the need for informed consent was waived by Kyoto University Graduate School and Faculty of Medicine and Kyoto University Hospital Ethics Committee. This study was carried out utilising the opt-out procedure described on our hospital website.

## Data Availability

The datasets generated during and/or analysed during the current study are available from the corresponding author upon reasonable request.
